# The effects of various diets on glycemic outcomes during pregnancy: A systematic review and network meta-analysis

**DOI:** 10.1371/journal.pone.0182095

**Published:** 2017-08-03

**Authors:** Vanessa Ha, Ashley J. Bonner, Jaynendr K. Jadoo, Joseph Beyene, Sonia S. Anand, Russell J. de Souza

**Affiliations:** 1 Department of Health Research Methods, Evidence, and Impact, Faculty of Health Sciences, McMaster University, Hamilton, Ontario, Canada; 2 Population Genomics Program, Chanchlani Research Centre, McMaster University, Hamilton, Ontario, Canada; 3 The Dalla Lana School of Public Health, Faculty of Medicine, University of Toronto, Toronto, ON, Canada; 4 The Hospital for Sick Children Research Institute, Toronto, ON, Canada; 5 Department of Medicine, Faculty of Health Sciences, McMaster University, Hamilton, Ontario, Canada; 6 Population Health Research Institute, Hamilton Health Sciences, McMaster University, Hamilton, Ontario, Canada; 7 General Hospital Campus, Hamilton, Ontario, Canada; Universidade de Sao Paulo, BRAZIL

## Abstract

**Aims:**

Evidence to support dietary modifications to improve glycemia during pregnancy is limited, and the benefits of diet beyond limiting gestational weight gain is unclear. Therefore, a systematic review and network meta-analysis of randomized trials was conducted to compare the effects of various common diets, stratified by the addition of gestational weight gain advice, on fasting glucose and insulin, hemoglobin A1c (Hb_A1c_), and homeostatic model assessment for insulin resistance (HOMA-IR) in pregnant women.

**Methods:**

MEDLINE, EMBASE, Cochrane database, and reference lists of published studies were searched through April 2017. Randomized trials directly comparing two or more diets for ≥2-weeks were eligible. Bayesian network meta-analysis was performed for fasting glucose. Owing to a lack of similar dietary comparisons, a standard pairwise meta-analysis for the other glycemic outcomes was performed. The certainty of the pooled effect estimates was assessed using the GRADE tool.

**Results:**

Twenty-one trials (1,865 participants) were included. In general, when given alongside gestational weight gain advice, fasting glucose improved in most diets compared to diets that gave gestational weight gain advice only. However, fasting glucose increased in high unsaturated or monounsaturated fatty acids diets. In the absence of gestational weight gain advice, fasting glucose improved in DASH-style diets compared to standard of care. Although most were non-significant, similar trends were observed for these same diets for the other glycemic outcomes. Dietary comparisons ranged from moderate to very low in quality of evidence.

**Conclusion/Interpretation:**

Alongside with gestational weight gain advice, most diets, with the exception of a high unsaturated or a high monounsaturated fatty acid diet, demonstrated a fasting glucose improvement compared with gestational weight gain advice only. When gestational weight gain advice was not given, the DASH-style diet appeared optimal on fasting glucose. However, a small number of trials were identified and most dietary comparisons were underpowered to detect differences in glycemic outcomes. Further studies that are high in quality and adequately powered are needed to confirm these findings.

**Registration:**

**PROSPERO** CRD42015026008

## Introduction

The need for implementation of effective dietary strategies in gestational diabetes mellitus (GDM) prevention and management has been emphasized by diabetes organizations [1–3]. Most women also prefer to not use medications to manage their diabetes risk during pregnancy [4].

One method of managing GDM risk is the use of dietary strategies. Data from individual randomized trials suggest benefits of dietary strategies in diabetes control [5–7]. The success of diet and lifestyle changes in managing type 2 diabetes mellitus (T2DM), some of its etiology shared with GDM, in high-risk patients further emphasize the importance of dietary strategies in GDM management [8]. Nonetheless, the evidence to support the application of dietary strategies to the treatment of GDM is lacking [1–3]. Further, a clear benefit for dietary strategies have not been demonstrated in recent meta-analyses [9, 10]. However, these analyses have usually been limited to single pair-wise dietary comparisons with a small number of participants. Furthermore, single pair-wise comparisons do not lend itself easily to determine if it is the most effective strategy amongst all the possible dietary strategies for GDM control.

The above concerns are reflected in current dietary guidelines for GDM prevention and management. Recommendations by the Canadian Diabetes Association (CDA) have not been updated in almost a decade and most are based on expert consensus, despite that dietary interventions are recommended as the first-line of therapy [3]. This has been echoed by the American Diabetes Association (ADA) and the National Institute of Health and Care Excellence (NICE) in the U.K., both of which claim no evidence-based recommendations can be made given the lack of high-quality research in this area [2, 11]. Although the importance of diet is acknowledged in GDM prevention and management, current dietary recommendations for GDM are sparse, and where it exists, is outdated or based on experts’ opinion [1–3].

Our goal in this study was to conduct a systematic review and network meta-analysis (NMA) of randomized trials to compare and rank the relative efficacy of various diets on glycemic outcomes in pregnant women with or without diabetes. Our analysis was stratified based on whether gestational weight gain (GWG) advice was given in addition to the dietary interventions so that the effects of diet can be isolated.

## Materials and methods

### Study protocol

The Cochrane Handbook for Systematic Reviews of Interventions (version 5.2) [12] and the Preferred Reporting Items for Systematic Reviews and Meta-Analyses (PRISMA) for network meta-analyses [13] was followed for analysis and reporting of results, respectively. The PRISMA checklist is provided in the supplemental material (**S1 File**). The protocol was registered with PROSPERO (CRD42015026008) (**S2 File**).

### Data sources and searches

MEDLINE, EMBASE, and Cochrane were searched up until April 2017 (**S1 Table**). A manual search of the references of the included studies was also conducted to identify additional eligible studies.

### Study selection

Each study identified by the electronic or manual search was screened by title and abstract to assess for inclusion by one reviewer (V.H.). Studies that passed the title/abstract screening were retrieved for full-text review. Eligible studies were randomized trials that examined the effect of one dietary intervention compared to another dietary intervention or standard of care on glycemic outcomes in pregnant women with or without diabetes and who were followed for at least two-weeks. A minimum of two-weeks of follow-up duration was chosen in accordance with diabetes guidelines which recommend that dietary therapy should be given for at least two-weeks before the use of insulin therapy [1–3]. Fasting glucose (FG) and insulin (FI), hemoglobin-A1c (Hb_A1c_)_,_ and homeostatic model assessment for insulin resistance (HOMA-IR) were glycemic outcomes of interest. No restriction was placed on language.

### Data extraction and quality assessment

Study characteristics and data from eligible studies were independently extracted by two reviewers (V.H. and J.K.J.). Extracted data included article citation, study design, participant characteristics, dietary interventions and macronutrient composition, level of feeding control, institution and country at which the study was conducted, study results, and statistical tests used. To ensure accuracy, extracted data were compared between the two reviewers and any discrepancies were resolved through consensus.

The quality of evidence for each dietary comparison was assessed using the Grading of Recommendations, Assessment, Development and Evaluation (GRADE) approach [14]. The overall quality of evidence for each dietary comparison was rated as high, moderate, low, or very low. Depending on the type of evidence in question, the starting point for GRADE assessment differed. Direct comparisons, where head-to-head comparisons from randomized trials were available, started at high quality of evidence and were downgraded based on the degree of study limitation, imprecision of pooled effect estimates, inconsistency of results, indirectness, and publication bias. First-order indirect comparisons, where two interventions had been individually compared against one common comparator but not with each other, started at the lower rating of the two dietary comparisons that made up the link and were downgraded based on evidence of intransitivity. Second and higher order indirect comparisons, where ≥2 common comparators were found between the two interventions being compared, were always rated as very low because of the distance between the two dietary interventions being compared.

### Statistical analysis

The network meta-analysis was conducted using R (version 3.2.0, R Project for Statistical Computing) with the *gemtc* and *rjags* packages, which interface with Just Another Gibbs Sampler (JAGS) software (version 3.4.0).

Prior to conducting the network meta-analysis, the assumptions of homogeneity and transitivity were assessed. Homogeneity, which reflects the degree of similarity between the effect estimates of each trial within the same dietary comparison, was assessed using Higgins criteria for I^2^ [12]. The I^2^ was chosen because it quantifies the degree of variation between trials that is due to inter-study heterogeneity and not by chance. Transitivity, which reflects the distribution of effect modifiers between trials, was assessed by examining the distribution of *a priori* effect modifiers for both direct and indirect dietary comparisons including stage of pregnancy (first, second, or third trimester), diagnosis of GDM (yes or no), pre-pregnancy body weight (as a continuous variable), and ethnicity (Europeans, Asians, Africans, or others).

A NMA for FG was performed. Relative effect estimates from the NMA are expressed as median differences (MeD) with 95% credible intervals (CrIs). MeD and their CrIs can be interpreted in the same manner as traditional mean differences (MD) with 95% confidence intervals (CIs). The FG achieved at the end of each dietary intervention for each included trial was extracted and pooled using the Bayesian fixed effects model, with a minimally informative prior distribution for relative treatment effects. A fixed effects model was chosen because it had a lower deviance information criterion (DIC) compared to the random effects model, suggesting a better model fit. Non-informative prior distributions were chosen for model parameters so that results were driven entirely by the reported data. Analyses were performed using Markov-Chain Monte-Carlo methods, a method that estimates the effect of each dietary comparison by simulation, using four chains with 200,000 iterations and thinning interval of ten, after a burn-in of 100,000. Convergence of the chains was assessed using the Gelman plot and diagnostic test [15]. Consistency of direct and indirect sources of evidence within the network was assessed using the node-splitting method [16]. Statistical significance was considered when the CrIs did not cross the line of no effect.

Surface Under the Cumulative Ranking (SUCRA) values were calculated to assist in determining the probability of a given dietary intervention as being the best overall among the interventions compared, but this does not necessarily reflect that the dietary intervention is good to treat with as other important clinical factors are not considered in the calculation (e.g. patient preferences, cost-effectiveness, etc.). The closer SUCRA is to 100, the more certain we are that it is the best overall and the closer it is to zero, the more certain we are that it is worst [17]. Ranks, cumulative ranks, and SUCRA values were considered as supplementary measures to the primary effect estimates for each dietary comparison because the former three measures are known to have substantive uncertainty [18].

Standard pair-wise meta-analyses for FI, Hb_A1c_, and HOMA-IR were performed because they lacked a common dietary comparator that connected them to a network plot. Results were expressed as MD with 95% CIs. The glycemic outcome achieved at the end of each dietary intervention for each included trial was extracted and pooled using the fixed effects model as there were <10 studies included per analysis. Significance was considered when p<0.05.

Analyses were stratified by whether advice regarding optimal weight gain during pregnancy was given in addition to the dietary intervention (“GWG advice”). Trials were considered to have given participants GWG advice if the investigators established energy requirements so that women would achieve appropriate GWG. Trials were grouped into “trials with GWG advice provided in both dietary arms” if the study was designed to include GWG advice in addition to the dietary interventions. In contrast, trials were grouped into “trials with no GWG advice” if no GWG advice was given at all. Finally, studies were grouped into “trials with GWG advice provided in one of the dietary arms” if only one of the dietary interventions included GWG advice but not the other. Studies, where GWG advice was given in only one of the dietary arm but not in the comparator, were not included in the NMA. Further, studies were not included in the NMA if they did not connect to the network plot due to a lack of a common comparator. A standard pairwise meta-analysis was performed for these types of studies.

## Results

### Literature search and study characteristics

**Fig 1:**
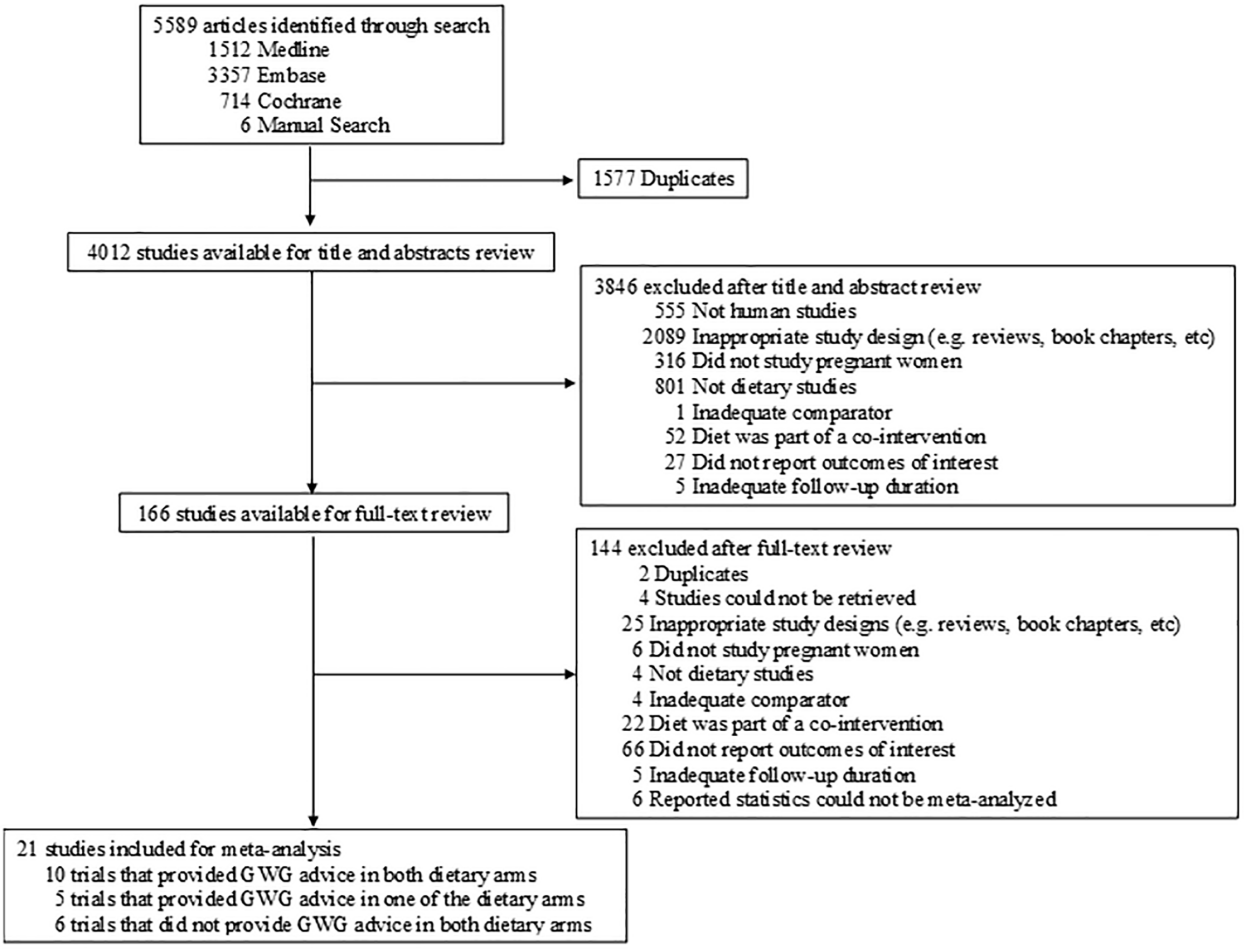
Flow of the literature search.

Of the 5589 studies that were identified, twenty-one studies were included (**Fig 1**) [5, 6, 19–37]. Ten trials were designed to include GWG advice in addition to the dietary intervention, five trials included GWG advice in only one of the dietary arms, and seven trials did not report giving any GWG advice in either arm.

Participants were predominantly young women (median = 30.6 years [interquartile range (IQR): 29.5 to 30.9 years]) in their second trimester at the start of the study (median = 24.4 weeks [IQR: 20.8 to 28.5 weeks]) with some degree of glucose intolerance (**S2 Table**). Most participants were considered overweight based on their pre-pregnancy BMI (median = 26.6 kg/m^2^ [IQR: 23.5, 27.7]). Smokers were included in one trial only (20% of included participants).

Overall, the baseline FG (median = 4.9 mmol/L [IQR: 4.7 to 5.0]) and Hb_A1c_ (median = 5.7% [IQR: 4.9 to 5.4%]) were within the normal range. The median baseline FI was 99.8 pmol/L (IQR: 63.8 to 135.2 pmol/L) and the median baseline HOMA-IR was 2.2 (IQR: 1.3 to 2.5).

Macronutrient composition was targeted in twenty-three dietary arms. Carbohydrate (CHO) intake was the focus of fifteen dietary arms (a low- glycemic index or load [GI and GL, respectively] diet in six arms, a high-fibre diet in three, a low-GI/GL and high-fibre in one, a low-CHO and low GI diet in two, and a low-CHO diet in three). Fat intake was the focus of four dietary arms (low fat in one arm, high monounsaturated fatty acid (MUFA) intake in one, and high unsaturated fat intake in two), a low-CHO and high fat diet in three dietary arms, and a high-fibre and low-fat diet in one dietary arm. Diets that targeted whole patterns of food consumption were the focus of fourteen dietary arms. The Dietary Approach to Stop Hypertension (DASH) diet was used in three dietary arms, healthy eating was used in two dietary arms, calorie restriction only was used in nine dietary arms. Standard of care, which were dietary arms with no dietary advice given or a standard macronutrient distribution (45–64% of energy from CHO: 10–35% of energy from protein: 20–35% of energy from fat) was followed, was used in seven dietary arms.

Six trials were conducted in North America (Canada two, U.S. three, and Mexico one), seven trials were conducted in Europe (Italy and Denmark two each, and Finland, Ireland, and Poland had one each), three trials were conducted in Australia, and six were conducted in Asia (Iran and China had three trials each). The median follow-up duration was 11.0 weeks (IQR: 7.1 to 14.8 weeks).

### Network assumptions

The assumptions of homogeneity and transitivity for NMAs were reasonably met. No evidence of inter-study heterogeneity was found between trials of dietary comparisons that did not provide GWG advice (I^2^ = 0%). Within trials that offered GWG advice, inter-study heterogeneity was low (range: 0 to 45.5%). Further, too few studies reported pre-pregnancy BMI (n = 8 trials) to assess whether the transitivity assumption was violated due to an imbalance on this characteristics across trials, but there was no evidence of an imbalanced distribution of effect modifiers for GDM diagnosis, ethnicity, and pregnancy stage.

### Trials with GWG advice provided in both dietary arms

#### Fasting glucose

**Fig 2:**
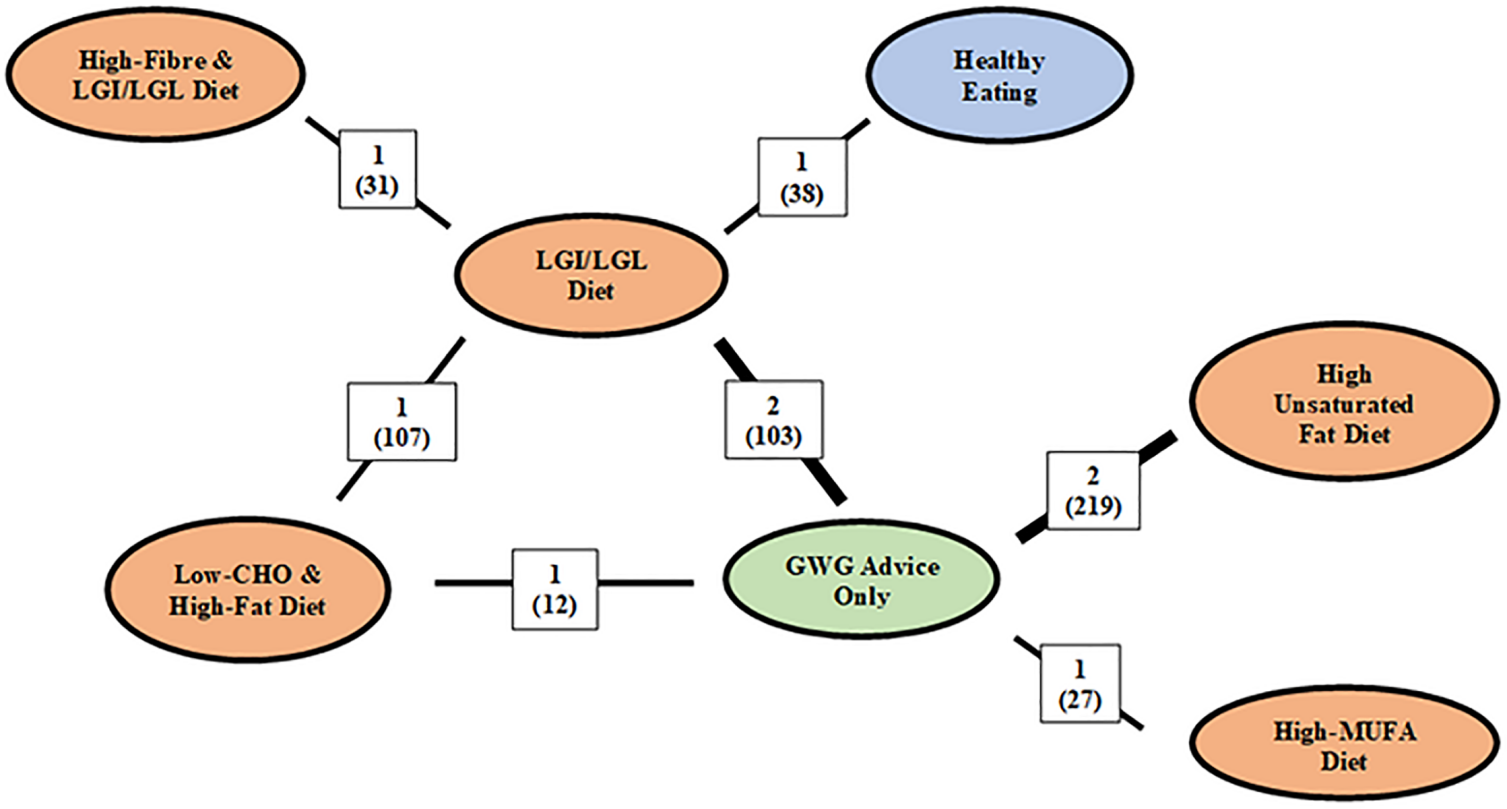
Network geometry of trials that provided GWG advice in both arms and reported fasting glucose. Abbreviations: CHO, carbohydrate; LGI, low-glycemic index; LGL, low-glycemic load; GWG, gestational weight gain; MUFA, monounsaturated fatty acids. The colors of each node correspond to a different diet class: orange node represents diets that targeted macronutrient intake, blue nodes represent diets that targeted overall healthy eating, and green nodes represent diets that targeted GWG. The numbers above each line joining two comparators correspond to the number of trials that compare the treatments with the number of included participants expressed in brackets. Thickness of line represent the number of studies included for that dietary comparison. Distances between nodes are not meaningful.

GWG advice was given in addition to dietary interventions and had FG reported in nine trials (**Fig 2**) [5, 21, 22, 25, 28, 31–35].

**Fig 3:**
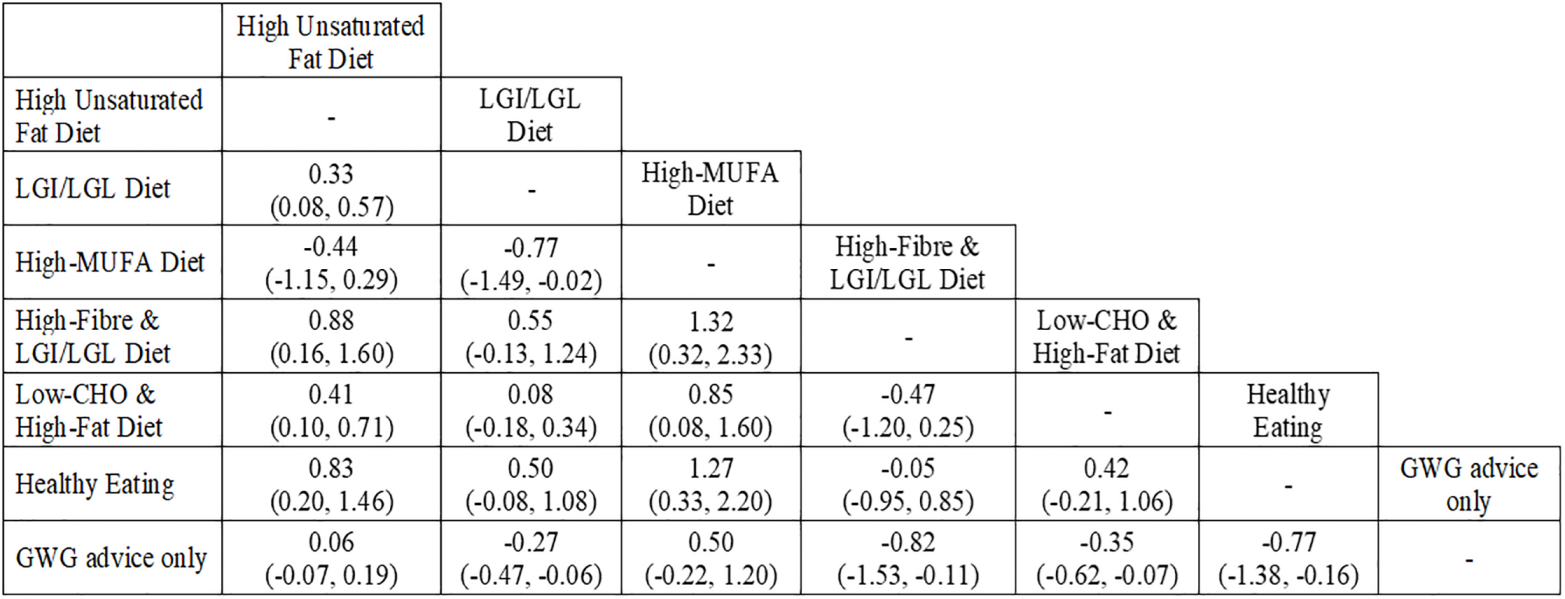
Difference in median effect with 95% credible intervals between diets given in addition to GWG advice on fasting glucose in mmol/l. Abbreviations: CHO, carbohydrate; LGI, low-glycemic index; LGL, low-glycemic load; GWG, gestational weight gain; MUFA, monounsaturated fatty acids. The value in each cell expresses the median difference and its 95% credible intervals between the dietary pattern in the column and the dietary pattern in the row (e.g. the median difference of the high-unsaturated fat diet compared to LGI/LGL diet is 0.33 mmol/L (95% CrIs = 0.08, 0.57 mmol/L).

Where direct comparisons were available, no between diet differences were observed (high unsaturated fat diets vs GWG advice only and high-MUFA diet vs GWG advice) (**Fig 3**). Using indirect comparisons, in general, FG increased in diets that modified fat quality intake compared with other diets. FG increase was observed in four out of the six dietary comparisons that prescribed a high unsaturated fat diet and three out of four dietary comparisons that involved a high-MUFA diet.

FG was improved when appropriate GWG advice was given alongside dietary advice compared with GWG advice only. FG reduction was observed in four of the six dietary comparisons, two of which were derived from mixed comparisons (LGI/LGL diets vs GWG advice only and low-CHO & high-fat diet vs GWG advice only) and the other two were derived from indirect comparisons (high-fibre & LGI/LGL vs GWG advice only and Healthy Eating vs GWG advice only).

The most effective diet to reduce FBG was the low-GI, high-fibre diet (SUCRA = 89.33%), followed by Healthy Eating (SUCRA = 88.17%), and then a low-CHO with a high-fat diet (SUCRA = 65.05%) (**S1 Fig**).

#### Other glycemic outcomes

A high-MUFA diet compared to GWG advice only increased HbA1c (MeD = 0.40% [95% CrIs: 0.12, 0.68]) (**S2 Fig**). No significant differences in Hb_A1c_, FI, and HOMA-IR were seen between pairs of any other diets (**S2–S4 Figs**).

#### Insulin therapy

In a *post-hoc* NMA analysis, based on an indirect comparison, the odds of progressing to insulin therapy to manage hyperglycemia during pregnancy was greater for a LGI diet than to a combined LGI and high-fibre diet (odds ratio [OR] = 5.92 [95% CrI: 1.20, 36.41]). No other diets were associated with the use of insulin therapy (data not shown).

### Trials with GWG advice provided in one of the dietary arms

A significant FI reduction was observed when comparing GWG advice to standard of care (MD = -25.00 pmol/L [95% CIs: -46.50, -3.50]) (**S7 Fig**). No significant FG (**S5 Fig**) or Hb_A1c_ (**S6 Fig)** effect was observed in any of the dietary comparisons.

### Trials with no GWG advice provided in both dietary arms

#### Fasting glucose

**Fig 4:**
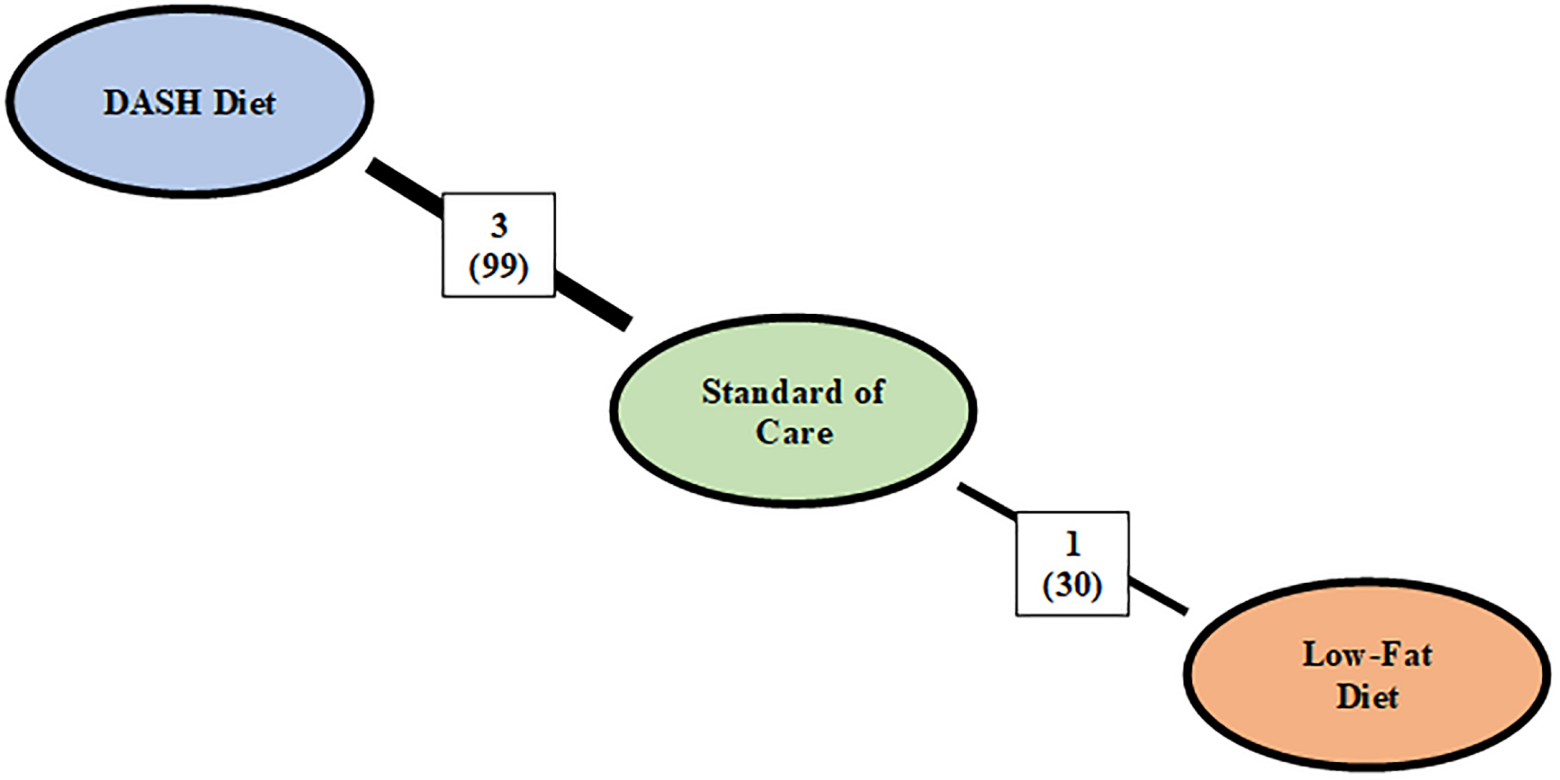
Network plot of trials that reported fasting glucose and did not provide gestational weight gain advice in both dietary arms. Abbreviations: DASH, Dietary Approach to Stop Hypertension. The colors of each nodep correspond to a different diet class: orange node represents diets that targeted macronutrient composition, blue represents diets that targeted food consumption, and green on weight gain advice. The number above each line correspond to the number of trials that compared the two diets with the number of included participants expressed in brackets.

Dietary interventions given with no GWG advice and had FG reported were identified in six trials (**Fig 4**) [23, 24, 27, 29, 30, 36].

**Fig 5:**
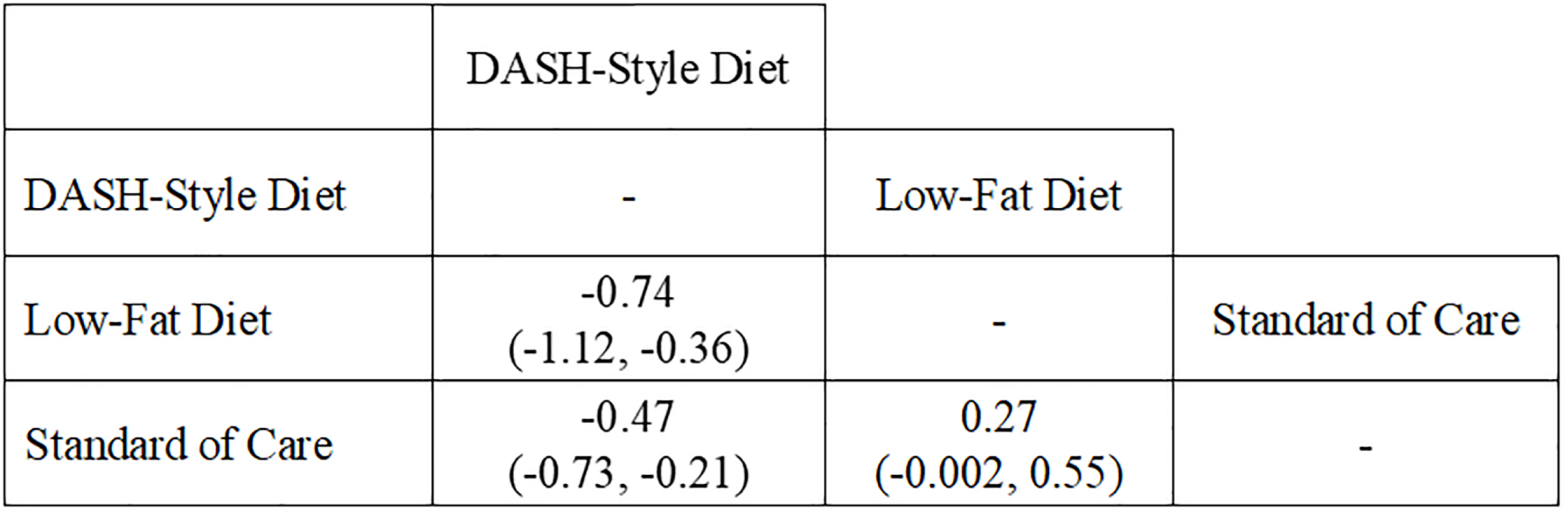
Effect of fasting glucose between diets in trials that did not provide gestational weight gain advice in both dietary arms. Abbreviations: DASH, Dietary Approach to Stop Hypertension. Fasting glucose is expressed in mmol/L. The value in each cell expresses the median difference (MeD) in fasting glucose with the 95% credible intervals (CrIs) in brackets between the diet in the column and the diet in the row (e.g. the MeD in fasting glucose between DASH-style diet compared to low-fat diet is -0.74 mmol/L (95% CrIs: -1.12, -0.36).

In the absence of GWG advice, an improvement in FG was found in DASH-style diet compared to other diets (**Fig 5**). FG was reduced for the DASH-style diet in an indirect comparison with low-fat diet (MeD = -0.74 mmol/L [95% CrIs: -1.12, -0.36]) and in a direct comparison with standard of care (MeD = -0.47 mmol/L [95% CrIs: -0.73, -0.21]). Further, a non-significant FG-effect was observed in a low-GI diet compared to a high-fibre diet in a study that was not analyzed as part of the NMA due to a lack of a common comparator (MD = -0.10 mmol/L [95% CIs: -0.38, 0.18]; p = 0.48) [27].

The most effective diet to reduce FG in the absence of GWG advice was the DASH-style diet (SUCRA = 66.7%), followed by standard of care (SUCRA = 32.5%), and low-fat diet (SUCRA = 0.88%) (**S4 Table**).

#### Other glycemic outcomes

There were no significant differences on HbA1c (**S8 Fig**), FI (**S9 Fig**), and HOMA-IR (**S10 Fig**) between diets with the exception of an insulin-reducing effect (MD = -47.60 pmol/L [95% CIs: -77.34, -17.86]; p = 0.002) and a HOMA-IR-reducing effect (MD = -1.90 [95% CIs: -3.08, -0.72]; p = 0.002) in a DASH-style diet compared to standard of care in the absence of GWG advice.

#### Insulin therapy

None of the dietary comparisons showed a significant association to start insulin therapy to manage hyperglycemia during pregnancy in our post-hoc NMA analysis (data not shown).

### GRADE- quality of evidence assessment

The quality of the evidence ranged from moderate to very low (**S3–S8 Tables**). Most comparisons were downgraded because of serious concerns regarding indirectness and/or imprecision.

## Discussion

We have systematically reviewed and conducted a network meta-analysis of randomized trials to assess the relative effectiveness of various diets on glycemic outcomes in women during pregnancy. Alongside with gestational weight gain advice, most diets, with the exception of a high unsaturated or a high monounsaturated fatty acid diet, demonstrated a fasting glucose improvement compared with gestational weight gain advice only. When gestational weight gain advice was not given, the DASH-style diet appeared optimal on fasting glucose. Similar trends were observed in the other glycemic outcomes.

The benefits of diets given in addition to GWG advice or standard of care on FG appeared modest, but we believe that these have important clinical relevance. Reductions in FG of 0.1 mmol/L in the Metformin in Gestational Diabetes Trial [38] and 0.3 mmol/L in a large RCT were observed when insulin was compared to anti-hyperglycemic medications in pregnant women [39]. Similar magnitudes of FG reductions were observed in our analysis, ranging from -0.27 to -0.77 mmol/L in trials with GWG advice and -0.47 to -0.74 mmol/L in trials with no GWG advice. This is particularly important during pregnancy as most women prefer dietary approaches to manage FG levels than the use of insulin therapy [4]. Furthermore, our findings build on existing dietary approaches for management of GDM which mostly focus on carbohydrate-counting or limiting caloric intake to manage GDM risk [1–3]. All our dietary comparisons that demonstrated a FG improvement emphasized on the consumption of high-quality (e.g., unrefined, minimally processed foods such as vegetables and fruits, whole grains), healthy foods, and minimizing low-quality foods (e.g., highly processed snack foods, refined grains, fried foods, and high-GI foods). Providing high-quality diets may be more effective to manage FG than GWG advice or standard of care only.

Both the quantity and quality of diet have been emphasized as equally important in the management of cardiometabolic risk [40]. Pregnancy is a time of heightened sensitivity and attention to food intake in most women [4], so the ability of healthcare providers to provide accurate and evidence-based advice increases the relevance of our findings. Although most diets that were given in addition to GWG advice demonstrated a FG reduction in our NMA, these same findings were not found in trials that had been specifically designed to assess if diets in addition to GWG advice would affect FG. Instead, a null FG-effect had been reported by these trials. This may, however, be due to the small number trials of such trials identified and included in our analysis.

High unsaturated fat intake has been found to be cardio-protective but there is uncertainty concerning its relationship with diabetes risk. Although meta-analyses have shown non-significant findings, a trend for increased type 2 diabetes mellitus (T2DM) risk have been noted for polyunsaturated fatty acid intakes (PUFAs), omega-3’s, and foods that are a source of these fatty acids such as fish and other seafood [41, 42]. High fat intake is linked to increased hepatic glucose production by reducing the ability of insulin to suppress endogenous glucose production [43]. Trials that were included in our analysis showed a positive correlation between unsaturated fat or MUFA intakes with PUFA intakes [31, 34]. Consistent with the above findings between PUFAs and T2DM, our analysis found that FG increased in diets that increased unsaturated or MUFA intakes.

Insulin therapy is usually initiated after two weeks if women cannot manage their GDM using diet therapy alone [2, 3]. No difference in the use of insulin therapy was found between diets in our analysis except for LGI diets compared to LGI with high-fibre diets. One interpretation of this finding is that the examined interventions (diets, GWG advice, and standard of care) were equally effective in preventing the use of insulin. We cannot, however, rule out the more likely possibility that trials achieved suboptimal dietary compliance (as reflected in our GRADE assessment) or that the dietary contrasts were not large enough to detect effects on insulin therapy use.

Several limitations were noted in the present study. First, our network meta-analysis included only RCTs which may have limited the number of available dietary comparisons. We had, however, decided not to include non-randomized studies because of concerns that these types of studies are more likely to introduce bias into the effect estimates because of confounding arising from the lack of randomization. A specific barrier to including both randomized and non-randomized studies in a network meta-analysis is that this practice would compromise the validity of our network by possibly violating two key assumptions: transitivity (the distribution of patient and study characteristics that are modifiers of treatment effect be sufficiently similar across studies) and as such, could possibly affect the consistency of the evidence (agreement of direct and indirect evidence for a given pair of treatments). Second, our certainty in the pooled effect estimates for each dietary comparison was moderate to very low. For our FG analysis, the quality of evidence was downgraded mostly due to poor (indirect) network connectivity between diets, small sample sizes, or both. For other glycemic outcomes, a lack of similar dietary comparisons precluded us from conducting a useful NMA. Furthermore, most dietary comparisons were under-powered to detect a difference in FG, HbA1c, FI, or HOMA-IR as we had found in our *post-hoc* analysis (data not shown). Third, most of our findings were derived from indirect comparisons rather than direct comparisons. Although we concluded that the assumption of transitivity was reasonably met for indirect comparisons within our study, we were not able to use the less-reported BMI to guide our assessments and as always, the case with indirect comparisons, minor, immeasurable effect-modifying characteristics could bias these estimates. Fourth, the generalizability of our results is limited. Most of the included trials were predominantly in young women in their second trimester who were already diagnosed with GDM. Therefore, it is unclear if the studied diets can prevent GDM per se. Certainly, however, based on our results, some diets appeared to be more effective in managing glycemic outcomes than others. Notwithstanding these limitations, many of the dietary comparisons in our analyses were designed to assess two dietary interventions that may benefit glycemic control; as such comparisons to a usual diet (e.g. typical North American/European non-therapeutic diet) were few and in this regard, a maintenance in glycemic control after intervention may be noteworthy.

## Conclusions

Alongside with gestational weight gain advice, most diets, with the exception of a high unsaturated or a monounsaturated fatty acid diet, demonstrated a fasting glucose improvement compared with gestational weight gain advice only. When gestational weight gain advice was not given, the DASH-style diet appeared optimal on fasting glucose. However, the number of trials is small and most were underpowered to detect differences in FG. To clarify the role of diets in glycemic management during pregnancy, data from larger, high-quality, and well-powered feeding trials of dietary approaches and high-quality prospective cohort studies are required. Nonetheless, diets, with the exception of ones that modify fat intake, may be useful as part of a strategy to improve FG.

## Supporting information

S1 Fig Rank of each diet that were given in addition to GWG advice as being the most effective in reducing fasting glucose. Abbreviations: CHO, carbohydrate; GWG, gestational weight gain; LGI, low glycemic index; LGL, low glycemic load; MUFA, monounsaturated fatty acids; SUCRA, surface under the cumulative ranking. The rankogram displays the probability of each diet achieving a particular rank and the SUCRA value reflects the probability of a given diet as being the most effective in reducing fasting glucose among all the diets being compared. The closer SUCRA is to 100, the more certain we are that it is the best overall and the closer it is to zero, the more certain we are that it is worst.
(DOCX)

S2 Fig Pair-wise meta-analyses of diets and Hb_A1c_ in trials where GWG advice was provided in both dietary arms. Abbreviations: CHO, carbohydrate; CI, confidence interval; GWG, gestational weight gain; HbA1c, hemoglobin A1c; LGI, low glycemic index; MD, mean differences; MUFA, monounsaturated fatty acids; n, sample size. Diet # 1 reflects the diet that is first mentioned before “vs” and diet #2 reflects the diet that comes after “vs”.
(DOCX)

S3 Fig Pair-wise meta-analyses of diets and fasting insulin in trials where GWG advice was provided in both dietary arms. Abbreviations: CHO, carbohydrate; CI, confidence interval; FI, fasting insulin; GWG, gestational weight gain; MD, mean differences; n, sample size. Diet # 1 reflects the diet that is first mentioned before “vs” and diet #2 reflects the diet that comes after “vs”.
(DOCX)

S4 Fig Pair-wise meta-analyses of diets and HOMA-IR in trials where GWG advice was provided in both dietary arms. Abbreviations: CHO, carbohydrate; CI, confidence interval; GWG, gestational weight gain; HOMA-IR, homeostatic model assessment for insulin resistance; MD, mean differences; n, sample size.
(DOCX)

S5 Fig Pair-wise meta-analysis of diets and fasting glucose in trials where GWG advice was provided in one of the dietary arms. Abbreviations: CHO, carbohydrate; CI, confidence interval; FG, fasting glucose; GWG, gestational weight gain; MD, mean differences; n, sample size.
(DOCX)

S6 Fig Pair-wise meta-analysis of diets and Hb_A1c_ in trials where GWG advice was provided in one of the dietary arms. Abbreviations: CHO, carbohydrate; CI, confidence interval; GWG, gestational weight gain; HbA1c, hemoglobin A1c; MD, mean differences; n, sample size.
(DOCX)

S7 Fig Pair-wise meta-analysis of diets and fasting insulin in trials where GWG advice was provided in one of the dietary arms. Abbreviations: CHO, carbohydrate; CI, confidence interval; FI, fasting insulin; GWG, gestational weight gain; MD, mean differences; n, sample size.
(DOCX)

S8 Fig Pair-wise meta-analysis of diets and Hb_A1c_ in trials with no GWG advice provided. Abbreviations: CI, confidence interval; DASH, Dietary Approach to Stop Hypertension; HbA1c, hemoglobin A1c; LGI, low glycemic index; MD, mean differences; n, sample size. Diet # 1 reflects the diet that is first mentioned before “vs” and diet #2 reflects the diet that comes after “vs”.
(DOCX)

S9 Fig Pair-wise meta-analysis of diets and fasting insulin in trials with no GWG advice provided. Abbreviations: CI, confidence interval; DASH, Dietary Approach to Stop Hypertension; FI, fasting insulin; LGI, low glycemic index; MD, mean differences; n, sample size. Diet # 1 reflects the diet that is first mentioned before “vs” and diet #2 reflects the diet that comes after “vs”.
(DOCX)

S10 Fig Pair-wise meta-analysis of diets and HOMA-IR in trials with no GWG advice provided. Abbreviations: CI, confidence interval; DASH, Dietary Approach to Stop Hypertension; FI, fasting insulin; LGI, low glycemic index; MD, mean differences; n, sample size. Diet # 1 reflects the diet that is first mentioned before “vs” and diet #2 reflects the diet that comes after “vs”.
(DOCX)

S1 Table Search strategy used to identify eligible studies. Search was conducted in November 2014 and updated in April 2015, February 2016, and April 2017.
(DOCX)

S2 Table Table of study characteristics. Abbreviations: "-," not reported; "~," calculated; BMI, body mass index; CHO, carbohydrate; DASH, Dietary Approach to Stop Hypertension; FG, fasting glucose; FI, fasting insulin; GDM, gestational diabetes mellitus; GWG, gestational weight gain; HbA1c, hemoglobin A1c; HOMA-IR, homeostatic model assessment-insulin resistance; IGT, Impaired Glucose Tolerance; LGI, low glycemic index; LGL, low glycemic load; MUFA, monounsaturated fatty acids; Ob, Obese; OW, overweight; T1DM, type 1 Diabetes; T2DM, type 2 diabetes; *All data expressed as mean ± SD unless otherwise noted. †Dietary definitions: DASH-style intake, diets rich in fruits, vegetables, whole grains, low-fat dairy products but low in saturated fats, cholesterol, refined grains, sweets, and sodium; GWG advice only, advice given to help women achieve optimal GWG; healthy eating, advice followed general healthy eating guidelines (e.g. Canada’s Food Guide); high-fat, >30% of energy came from fat; high-fibre, >30 g/d of dietary fibre; high unsaturated fat, increased in unsaturated fat intake compared to no intervention; low-CHO, <45% energy came from CHO; low-fat, <20% of energy from fat; LGI, low glycemic index; LGL, low glycemic load; standard of care, no dietary advice given or macronutrient intake was 45–56% energy from CHO: 10–35% energy from protein: 20–35% energy from fat. ‡Body weight was reported when BMI is unavailable. δThe number of active smokers during pregnancy. Counts were reported with the percentage of the total participants as smokers reported in brackets. ¶The gestational week at which the participants started the dietary intervention. §Baseline characteristics were based on the number of randomised participants for Grant et al n = 43, Laitenin et al n = 171, Rae et al n = 124, and Valentini et al n = 781. **All foods were provided
(DOCX)

S3 Table Quality of the evidence in the direct dietary comparisons in the fasting glucose analysis. Abbreviations: CHO, carbohydrate; CrI, credible intervals; DASH, Dietary Approach to Stop Hypertension; FG, fasting glucose; GWG, gestational weight gain; LGI, low glycemic index; LGL, low glycemic load; MeD, median difference; MUFA, monounsaturated fatty acids; n, sample size. ^a^Dietary comparison was downgraded because the attrition rate of the included trial was considered to have high risk of bias. ^b^Inconsistency could not be assessed because only one trial was included. ^c^Although there was evidence of moderate inter-study heterogeneity (I^2^ = 44.6%), this was not a cause of concern because the credible intervals of the two included trials substantially overlapped one another and their point estimate laid on the same side of the line of no effect. ^d^No evidence of inter-study heterogeneity (I^2^ = 0%). ^e^The included trial(s) failed to achieve its dietary goals and therefore, the contrast of the dietary interventions may be too small to affect FG. ^f^Optimal information size (OIS) was not met. ^g^The effect estimate crosses the minimally important difference (MID) of ±0.5 mmol/L. ^h^Publication bias could not be assessed because there were <10 included trials.
(DOCX)

S4 Table Quality of the evidence in the indirect dietary comparisons in the fasting glucose analysis. Abbreviations: CHO, carbohydrate; CrI, credible intervals; DASH, Dietary Approach to Stop Hypertension; FG, fasting glucose; GWG, gestational weight gain; LGI, low glycemic index; LGL, low glycemic load; MeD, median difference; MUFA, monounsaturated fatty acids. ^a^There were important differences in GDM status and at the trimester in which the dietary interventions began between the two first order links. ^b^There were important differences in GDM status, ethnicity, and at the trimester in which the dietary interventions began between the two first order links. ^c^Quality of comparisons was assumed as very low because the link order is ≥2. ^d^There were important differences in ethnicity and at the trimester in which the dietary interventions began between the two first order links. ^e^There were important differences at the trimester in which the dietary interventions began between the two first order links. ^f^There were important differences in ethnicity between the two first order links. ^g^There were important differences in pre-pregnancy BMI and at the trimester in which the dietary interventions began between the two first order links.
(DOCX)

S5 Table Quality of evidence in the mixed dietary comparisons in the fasting glucose analysis. Abbreviations: CHO, carbohydrate; CIs, confidence intervals; DASH, Dietary Approach to Stop Hypertension; GWG, gestational weight gain; HbA1c, hemoglobin A1c; LGI, low glycemic index; LGL, low glycemic load; MD, mean difference; MUFA, monounsaturated fatty acids; n, sample size. Abbreviations: CHO, carbohydrate; CrI, credible intervals; FG, fasting glucose; GWG, gestational weight gain; LGI, low glycemic index; LGL, low glycemic load; MeD, median difference.
(DOCX)

S6 Table Quality of the evidence in the direct dietary comparisons in the HbA1c analysis. ^a^Inconsistency could not be assessed because only one trial was included. ^b^There was evidence of high inter-study heterogeneity (I^2^ = 88%). Further the two included trials showed different effects, one showed protection and the other showed null. ^c^The included trial(s) failed to achieve its dietary goals and therefore, the contrast of the dietary interventions may be too small to affect HbA1c. ^d^The effect estimate crosses the minimally important difference (MID) of ±0.3%. ^e^Optimal information size (OIS) was not met. ^f^Publication bias could not be assessed because there were <10 included trials. ^g^No evidence of inter-study heterogeneity (I^2^ = 0%).
(DOCX)

S7 Table Quality of the evidence in the direct dietary comparisons in the fasting insulin analysis. Abbreviations: CHO, carbohydrate; CIs, confidence intervals; DASH, Dietary Approach to Stop Hypertension; FI, fasting insulin; GWG, gestational weight gain; LGI, low glycemic index; MD, mean difference; MUFA, monounsaturated fatty acids; n, sample size. ^a^Dietary comparison was downgraded because the attrition rate of the included trial was considered to have high risk of bias. ^b^Inconsistency could not be assessed because only one trial was included. ^c^No evidence of inter-study heterogeneity (I^2^ = 0%). ^d^The included trial(s) failed to achieve its dietary goals and therefore, the contrast of the dietary interventions may be too small to affect FI. ^e^The effect estimate crosses the minimally important difference (MID) of ±0.5 pmol/L. ^f^Optimal information size (OIS) was not met. ^g^Publication bias could not be assessed because there were <10 included trials.
(DOCX)

S8 Table Quality of the evidence in the direct dietary comparisons in the HOMA-IR analysis. Abbreviations: CHO, carbohydrate; CIs, confidence intervals; DASH, Dietary Approach to Stop Hypertension; GWG, gestational weight gain; HOMA-IR, homeostatic model assessment for insulin resistance; LGI, low glycemic index; MD, mean difference; n, sample size. ^a^Inconsistency could not be assessed because only one trial was included. ^b^The effect estimate crosses the minimally important difference (MID) of ±1 unit. ^c^Optimal information size (OIS) was not met. ^d^Publication bias could not be assessed because there were <10 included trials. ^e^No evidence of inter-study heterogeneity (I^2^ = 0%). ^f^The included trial failed to achieve its dietary goals and therefore, the contrast of the dietary interventions may be too small to affect HOMA-IR.
(DOCX)

S1 File PRISMA checklist. (DOCX)

S2 File Prospero protocol registration. (PDF)
