# Supplementary material for: The effects of various diets on glycemic outcomes during pregnancy: A systematic review and network meta-analysis
Source: PLoS One. 2017 Aug 3;12(8):e0182095. doi: 10.1371/journal.pone.0182095 (PMC5542432; doi:10.1371/journal.pone.0182095)
Supplement: S1 Fig — Abbreviations: CHO, carbohydrate; GWG, gestational weight gain; LGI, low glycemic index; LGL, low glycemic load; MUFA, monounsaturated fatty acids; SUCRA, surface under the cumulative ranking. The rankogram displays the probability of each diet achieving a particular rank and the SUCRA value reflects the probability of a given diet as being the most effective in reducing fasting glucose among all the diets being compared. The closer SUCRA is to 100, the more certain we are that it is the best overall and the closer it is to zero, the more certain we are that it is worst. (DOCX) [file pone.0182095.s001.docx]

**Figure S1. Rank of each diet that were given in addition to GWG advice as being the most effective in reducing fasting glucose.**

**Abbreviations:** CHO, carbohydrate; GWG, gestational weight gain; LGI, low glycemic index; LGL, low glycemic load; MUFA, monounsaturated fatty acids; SUCRA, surface under the cumulative ranking.

The rankogram displays the probability of each diet achieving a particular rank and the SUCRA value reflects the probability of a given diet as being the most effective in reducing fasting glucose among all the diets being compared. The closer SUCRA is to 100, the more certain we are that it is the best overall and the closer it is to zero, the more certain we are that it is worst.
