# Supplementary material for: The effects of various diets on glycemic outcomes during pregnancy: A systematic review and network meta-analysis
Source: PLoS One. 2017 Aug 3;12(8):e0182095. doi: 10.1371/journal.pone.0182095 (PMC5542432; doi:10.1371/journal.pone.0182095)
Supplement: S2 Fig — Abbreviations: CHO, carbohydrate; CI, confidence interval; GWG, gestational weight gain; HbA1c, hemoglobin A1c; LGI, low glycemic index; MD, mean differences; MUFA, monounsaturated fatty acids; n, sample size. Diet # 1 reflects the diet that is first mentioned before “vs” and diet #2 reflects the diet that comes after “vs”. (DOCX) [file pone.0182095.s002.docx]

**Figure S2. Pair-wise meta-analyses of diets and Hb_A1c_ in trials where GWG advice was provided in both dietary arms.***

**Abbreviations:** CHO, carbohydrate; CI, confidence interval; GWG, gestational weight gain; Hb_A1c_, hemoglobin A1c; LGI, low glycemic index; MD, mean differences; MUFA, monounsaturated fatty acids; *n*, sample size.

*****Diet # 1 reflects the diet that is first mentioned before “vs” and diet #2 reflects the diet that comes after “vs”.
