# Supplementary material for: The effects of various diets on glycemic outcomes during pregnancy: A systematic review and network meta-analysis
Source: PLoS One. 2017 Aug 3;12(8):e0182095. doi: 10.1371/journal.pone.0182095 (PMC5542432; doi:10.1371/journal.pone.0182095)
Supplement: S4 Fig — Abbreviations: CHO, carbohydrate; CI, confidence interval; GWG, gestational weight gain; HOMA-IR, homeostatic model assessment for insulin resistance; MD, mean differences; n, sample size. (DOCX) [file pone.0182095.s004.docx]

**Figure S4. Pair-wise meta-analyses of diets and HOMA-IR in trials where GWG advice was provided in both dietary arms.**

**Abbreviations:** CHO, carbohydrate; CI, confidence interval; GWG, gestational weight gain; HOMA-IR, homeostatic model assessment for insulin resistance; MD, mean differences; *n*, sample size.
