# Supplementary material for: The effects of various diets on glycemic outcomes during pregnancy: A systematic review and network meta-analysis
Source: PLoS One. 2017 Aug 3;12(8):e0182095. doi: 10.1371/journal.pone.0182095 (PMC5542432; doi:10.1371/journal.pone.0182095)
Supplement: S5 Fig — Abbreviations: CHO, carbohydrate; CI, confidence interval; FG, fasting glucose; GWG, gestational weight gain; MD, mean differences; n, sample size. (DOCX) [file pone.0182095.s005.docx]

**Figure S5. Pair-wise meta-analysis of diets and fasting glucose in trials where GWG advice was provided in one of the dietary arms.**

**Abbreviations:** CHO, carbohydrate; CI, confidence interval; FG, fasting glucose; GWG, gestational weight gain; MD, mean differences; *n*, sample size.
