# Supplementary material for: The effects of various diets on glycemic outcomes during pregnancy: A systematic review and network meta-analysis
Source: PLoS One. 2017 Aug 3;12(8):e0182095. doi: 10.1371/journal.pone.0182095 (PMC5542432; doi:10.1371/journal.pone.0182095)
Supplement: S9 Fig — Abbreviations: CI, confidence interval; DASH, Dietary Approach to Stop Hypertension; FI, fasting insulin; LGI, low glycemic index; MD, mean differences; n, sample size. Diet # 1 reflects the diet that is first mentioned before “vs” and diet #2 reflects the diet that comes after “vs”. (DOCX) [file pone.0182095.s009.docx]

**Figure S9. Pair-wise meta-analysis of diets and fasting insulin in trials with no GWG advice provided.***

**Abbreviations:** CI, confidence interval; DASH, Dietary Approach to Stop Hypertension; FI, fasting insulin; LGI, low glycemic index; MD, mean differences; *n*, sample size.

*Diet # 1 reflects the diet that is first mentioned before “vs” and diet #2 reflects the diet that comes after “vs”.
