# Supplementary material for: The effects of various diets on glycemic outcomes during pregnancy: A systematic review and network meta-analysis
Source: PLoS One. 2017 Aug 3;12(8):e0182095. doi: 10.1371/journal.pone.0182095 (PMC5542432; doi:10.1371/journal.pone.0182095)
Supplement: S1 Table — Search was conducted in November 2014 and updated in April 2015, February 2016, and April 2017. (DOCX) [file pone.0182095.s011.docx]

**Table S1.** **Search strategy used to identify eligible studies.***

| **DATABASE** | **SEARCH DATE** | **SEARCH STRATEGY** |
| --- | --- | --- |
| Medline | 1946  to  April week 4 2015 | 1. pregnant women/ OR pregnan*.tw. OR prenatal care/ OR prenatal.tw. OR maternal.tw. OR expectant mother*.tw. 2. exp diet/ OR exp dietary supplements/ OR exp diet therapy/ OR diet*.tw. OR exp food/ OR exp food habits/ OR exp food quality/ OR food*.tw. OR nutri*.tw. OR nutritional status/ 3. 1 AND 2 4. exp maternal nutritional physiological phenomena/ 5. 3 OR 4 6. exp diabetes, gestational/ OR gestational diabetes.tw. OR glucose tolerance test/ OR glucose tolerance*.tw. OR OGTT OR glycaem*.tw. OR glycem*.tw. 7. 5 AND 6 |

*Search was conducted in November 2014 and updated in April 2015, February 2016, and April 2017.
