# Supplementary material for: The effects of various diets on glycemic outcomes during pregnancy: A systematic review and network meta-analysis
Source: PLoS One. 2017 Aug 3;12(8):e0182095. doi: 10.1371/journal.pone.0182095 (PMC5542432; doi:10.1371/journal.pone.0182095)
Supplement: S2 Table — Abbreviations: "-," not reported; "~," calculated; BMI, body mass index; CHO, carbohydrate; DASH, Dietary Approach to Stop Hypertension; FG, fasting glucose; FI, fasting insulin; GDM, gestational diabetes mellitus; GWG, gestational weight gain; HbA1c, hemoglobin A1c; HOMA-IR, homeostatic model assessment-insulin resistance; IGT, Impaired Glucose Tolerance; LGI, low glycemic index; LGL, low glycemic load; MUFA, monounsaturated fatty acids; Ob, Obese; OW, overweight; T1DM, type 1 Diabetes; T2DM, type 2 diabetes; *All data expressed as mean ± SD unless otherwise noted. †Dietary definitions: DASH-style intake, diets rich in fruits, vegetables, whole grains, low-fat dairy products but low in saturated fats, cholesterol, refined grains, sweets, and sodium; GWG advice only, advice given to help women achieve optimal GWG; healthy eating, advice followed general healthy eating guidelines (e.g. Canada’s Food Guide); high-fat, >30% of energy came from fat; high-fibre, >30 g/d of dietary fibre; high unsaturated fat, increased in unsaturated fat intake compared to no intervention; low-CHO, <45% energy came from CHO; low-fat, <20% of energy from fat; LGI, low glycemic index; LGL, low glycemic load; standard of care, no dietary advice given or macronutrient intake was 45–56% energy from CHO: 10–35% energy from protein: 20–35% energy from fat. ‡Body weight was reported when BMI is unavailable. δThe number of active smokers during pregnancy. Counts were reported with the percentage of the total participants as smokers reported in brackets. ¶The gestational week at which the participants started the dietary intervention. §Baseline characteristics were based on the number of randomised participants for Grant et al n = 43, Laitenin et al n = 171, Rae et al n = 124, and Valentini et al n = 781. **All foods were provided (DOCX) [file pone.0182095.s012.docx]

**Table S2.** **Table of study characteristics.*** †

**Table S2. Table of study characteristics.*** †

Abbreviations: "-," not reported; "~," calculated; BMI, body mass index; CHO, carbohydrate; DASH, Dietary Approach to Stop Hypertension; FG, fasting glucose; FI, fasting insulin; GDM, gestational diabetes mellitus; GWG, gestational weight gain; Hb_A1c_, hemoglobin A1c; HOMA-IR, homeostatic model assessment-insulin resistance; IGT, Impaired Glucose Tolerance; LGI, low glycemic index; LGL, low glycemic load; MUFA, monounsaturated fatty acids; Ob, Obese; OW, overweight; T1DM, type 1 Diabetes; T2DM, type 2 diabetes.

*All data expressed as mean ± SD unless otherwise noted.

†Dietary definitions: DASH-style intake, diets rich in fruits, vegetables, whole grains, low-fat dairy products but low in saturated fats, cholesterol, refined grains, sweets, and sodium; GWG advice only, advice given to help women achieve optimal GWG; healthy eating, advice followed general healthy eating guidelines (e.g. Canada’s Food Guide); high-fat, >30% of energy came from fat; high-fibre, >30 g/d of dietary fibre; high unsaturated fat, increased in unsaturated fat intake compared to no intervention; low-CHO, <45% energy came from CHO; low-fat, <20% of energy from fat; LGI, low glycemic index; LGL, low glycemic load; standard of care, no dietary advice given or macronutrient intake was 45-56% energy from CHO: 10-35% energy from protein: 20-35% energy from fat.

‡Body weight was reported when BMI is unavailable.

δThe number of active smokers during pregnancy. Counts were reported with the percentage of the total participants as smokers reported in brackets.

¶The gestational week at which the participants started the dietary intervention.

§Baseline characteristics were based on the number of randomised participants for Grant et al n=43, Laitenin et al n= 171, Rae et al n= 124, and Valentini et al n= 781.

**All foods were provided
