# Supplementary material for: The effects of various diets on glycemic outcomes during pregnancy: A systematic review and network meta-analysis
Source: PLoS One. 2017 Aug 3;12(8):e0182095. doi: 10.1371/journal.pone.0182095 (PMC5542432; doi:10.1371/journal.pone.0182095)
Supplement: S3 Table — Abbreviations: CHO, carbohydrate; CrI, credible intervals; DASH, Dietary Approach to Stop Hypertension; FG, fasting glucose; GWG, gestational weight gain; LGI, low glycemic index; LGL, low glycemic load; MeD, median difference; MUFA, monounsaturated fatty acids; n, sample size. aDietary comparison was downgraded because the attrition rate of the included trial was considered to have high risk of bias. bInconsistency could not be assessed because only one trial was included. cAlthough there was evidence of moderate inter-study heterogeneity (I2 = 44.6%), this was not a cause of concern because the credible intervals of the two included trials substantially overlapped one another and their point estimate laid on the same side of the line of no effect. dNo evidence of inter-study heterogeneity (I2 = 0%). eThe included trial(s) failed to achieve its dietary goals and therefore, the contrast of the dietary interventions may be too small to affect FG. fOptimal information size (OIS) was not met. gThe effect estimate crosses the minimally important difference (MID) of ±0.5 mmol/L. hPublication bias could not be assessed because there were <10 included trials. (DOCX) [file pone.0182095.s013.docx]

**Table S3. Quality of the evidence in the direct dietary comparisons in the fasting glucose analysis.**

| **Dietary Comparison** | **No of trials  (*n* participants)** | **FG, mmol/L**  **MeD**  **(95% CrIs)** | **Risk of Bias** | **Consistency** | **Directness** | **Precision** | **Publication**  **Bias** | **Quality**  **of Evidence** |
| --- | --- | --- | --- | --- | --- | --- | --- | --- |
| **GWG advice provided in both dietary arms** | | | | | | | | |
| LGI/LGL diet vs  Low-CHO & High-fat diet | 1  (107) | -0.10  (-0.43, 0.22) | 0 | 0^b^ | -2^e^ | -1^f^ | 0^h^ | **⊕⭘⭘⭘**  **VERY LOW** |
| LGI/LGL diet vs  High-fibre & LGI/LGL diet | 1  (31) | 0.55  (-0.13, 1.24) | -1^a^ | 0^b^ | 0 | -1^f,g^ | 0^h^ | **⊕⊕⭘⭘**  **LOW** |
| LGI/LGL diet vs  Healthy eating | 1  (38) | 0.50  (-0.08, 1.08) | 0 | 0^b^ | 0 | -1^f,g^ | 0^h^ | **⊕⊕⊕⭘**  **MODERATE** |
| LGI/LGL diet vs  GWG advice only | 2  (103) | -0.18  (-0.41, 0.04) | 0 | 0^c^ | -2^e^ | -1^f^ | 0^h^ | **⊕⭘⭘⭘**  **VERY LOW** |
| Low-CHO & High-fat diet vs  GWG advice only | 1  (12) | -0.60  (-1.00, -0.21) | 0 | 0^b^ | 0 | -1^f,g^ | 0^h^ | **⊕⊕⊕⭘**  **MODERATE** |
| High unsaturated fat diet vs  GWG advice only | 2  (219) | 0.06  (-0.07, 0.19) | 0 | 0^d^ | -1^e^ | -1^f^ | 0^h^ | **⊕⊕⭘⭘**  **LOW** |
| High-MUFA diet vs  GWG advice only | 1  (27) | 0.50  (-0.22, 1.20) | 0 | 0^b^ | 0 | -1^f,g^ | 0^h^ | **⊕⊕⊕⭘**  **MODERATE** |
| **GWG advice provided in one of the dietary arms** | | | | | | | | |
| GWG advice vs  Standard of care | 1  (300) | -0.66  (-1.31, -0.01) | -1^a^ | 0^b^ | 0 | -1^f,g^ | 0^h^ | **⊕⊕⭘⭘**  **LOW** |
| Low-CHO diet & GWG advice vs  Low-CHO diet | 1  (124) | 0.10  (-0.10, 0.30) | 0 | 0^b^ | 0 | -1^f^ | 0^h^ | **⊕⊕⊕⭘**  **MODERATE** |
| **GWG advice not provided in any of the dietary arms** | | | | | | | | |
| DASH-style diet vs  Standard of dare | 3  (99) | -0.47  (-0.73, -0.21) | 0 | 0 | 0 | -1^f,g^ | 0^h^ | **⊕⊕⊕⭘**  **MODERATE** |
| Low-fat diet vs  Standard of care | 1  (30) | 0.27 (-0.002, 0.55) | 0 | 0^b^ | 0 | -1^f,g^ | 0^h^ | **⊕⊕⊕⭘**  **MODERATE** |
| LGI diet vs  High-fibre diet | 1  (92) | -0.10  (-0.38, 0.18) | 0 | 0^b^ | -2^e^ | -1^f^ | 0^h^ | **⊕⭘⭘⭘**  **VERY LOW** |

**Abbreviations:** CHO, carbohydrate; CrI, credible intervals; DASH, Dietary Approach to Stop Hypertension; FG, fasting glucose; GWG, gestational weight gain; LGI, low glycemic index; LGL, low glycemic load; MeD, median difference; MUFA, monounsaturated fatty acids; *n*, sample size.

^a^Dietary comparison was downgraded because the attrition rate of the included trial was considered to have high risk of bias.

^b^Inconsistency could not be assessed because only one trial was included.

^c^Although there was evidence of moderate inter-study heterogeneity (I^2^= 44.6%), this was not a cause of concern because the credible intervals of the two included trials substantially overlapped one another and their point estimate laid on the same side of the line of no effect.

^d^No evidence of inter-study heterogeneity (I^2^= 0%).

^e^The included trial(s) failed to achieve its dietary goals and therefore, the contrast of the dietary interventions may be too small to affect FG.

^f^Optimal information size (OIS) was not met.

^g^The effect estimate crosses the minimally important difference (MID) of ±0.5 mmol/L.

^h^Publication bias could not be assessed because there were <10 included trials
