# Supplementary material for: The effects of various diets on glycemic outcomes during pregnancy: A systematic review and network meta-analysis
Source: PLoS One. 2017 Aug 3;12(8):e0182095. doi: 10.1371/journal.pone.0182095 (PMC5542432; doi:10.1371/journal.pone.0182095)
Supplement: S4 Table — Abbreviations: CHO, carbohydrate; CrI, credible intervals; DASH, Dietary Approach to Stop Hypertension; FG, fasting glucose; GWG, gestational weight gain; LGI, low glycemic index; LGL, low glycemic load; MeD, median difference; MUFA, monounsaturated fatty acids. aThere were important differences in GDM status and at the trimester in which the dietary interventions began between the two first order links. bThere were important differences in GDM status, ethnicity, and at the trimester in which the dietary interventions began between the two first order links. cQuality of comparisons was assumed as very low because the link order is ≥2. dThere were important differences in ethnicity and at the trimester in which the dietary interventions began between the two first order links. eThere were important differences at the trimester in which the dietary interventions began between the two first order links. fThere were important differences in ethnicity between the two first order links. gThere were important differences in pre-pregnancy BMI and at the trimester in which the dietary interventions began between the two first order links. (DOCX) [file pone.0182095.s014.docx]

**Table S4.** **Quality of the evidence in the indirect dietary comparisons in the fasting glucose analysis.**

| **Dietary Comparison** | **FG, mmol/L**  **MeD (95% CrIs)** | **Quality  of First-Order Link** | **Similarity** | **Overall  Quality of Evidence** |
| --- | --- | --- | --- | --- |
| **GWG advice provided in both dietary arms** | | | | |
| High unsaturated fat diet vs  LGI/LGL diet | 0.33  (0.08, 0.57) | Very Low | 0^a^ | **⊕⭘⭘⭘**  **VERY LOW** |
| High unsaturated fat diet vs  High-MUFA diet | -0.44 (-1.15, 0.29) | Low | -1^b^ | **⊕⭘⭘⭘**  **VERY LOW** |
| High unsaturated fat diet vs  High-fibre & LGI/LGL diet | 0.88 (0.16, 1.60) | -^c^ | - | **⊕⭘⭘⭘**  **VERY LOW** |
| High unsaturated fat diet vs  Low-CHO & high-fat diet | 0.41 (0.10, 0.71) | Low | -1^a^ | **⊕⭘⭘⭘**  **VERY LOW** |
| High unsaturated fat diet vs  Healthy eating | 0.83 (0.20, 1.46) | -^c^ | - | **⊕⭘⭘⭘**  **VERY LOW** |
| LGI/LGL diet vs  High-MUFA diet | -0.77 (-1.49, -0.02) | Very Low | -1^d^ | **⊕⭘⭘⭘**  **VERY LOW** |
| LGI/LGL diet vs  Low-CHO & high-fat diet | 0.42  (-0.03, 0.86) | Very Low | -1^e^ | **⊕⭘⭘⭘**  **VERY LOW** |
| LGI/LGL diet vs  GWG advice only | -0.71  (-1.20, -0.20) | Very Low | -1^e^ | **⊕⭘⭘⭘**  **VERY LOW** |
| High-MUFA diet vs  High-fibre & LGI/LGL diet | 1.32 (0.32, 2.33) | -^c^ | - | **⊕⭘⭘⭘**  **VERY LOW** |
| High-MUFA diet vs  Low-CHO & high-fat diet | 0.85 (0.08, 1.60) | Moderate | 0 | **⊕⊕⊕⭘**  **MODERATE** |
| High-MUFA diet vs  Healthy eating | 1.27 (0.33, 2.20) | -^c^ | - | **⊕⭘⭘⭘**  **VERY LOW** |
| High-fibre & LGI/LGL diet vs  Low-CHO & high-fat diet | -0.47 (-1.20, 0.25) | Very Low | 0 | **⊕⭘⭘⭘**  **VERY LOW** |
| High-fibre & LGI/LGL diet vs  Healthy eating | -0.05 (-0.95, 0.85) | Low | 0 | **⊕⊕⭘⭘**  **LOW** |
| High-fibre & LGI/LGL diet vs  GWG advice only | -0.82 (-1.53, -0.11) | Very Low | -1^f^ | **⊕⭘⭘⭘**  **VERY LOW** |
| Low-CHO & high-fat diet vs  Healthy eating | 0.42 (-0.21, 1.06) | Very Low | 0 | **⊕⭘⭘⭘**  **VERY LOW** |
| Low-CHO & high-fat diet vs  GWG advice only | -0.08  (-0.48, 0.32) | Very Low | -1^g^ | **⊕⭘⭘⭘**  **VERY LOW** |
| Healthy eating vs  GWG advice only | -0.77 (-1.38, -0.16) | Very Low | 0 | **⊕⭘⭘⭘**  **VERY LOW** |
| **GWG advice not provided in any of the dietary arms** | | | | |
| DASH-style diet vs  Low-fat diet | -0.74  (-1.12, -0.36) | Moderate | 0 | **⊕⊕⊕⭘**  **MODERATE** |

**Abbreviations:** CHO, carbohydrate; CrI, credible intervals; DASH, Dietary Approach to Stop Hypertension; FG, fasting glucose; GWG, gestational weight gain; LGI, low glycemic index; LGL, low glycemic load; MeD, median difference; MUFA, monounsaturated fatty acids.

^a^There were important differences in GDM status and at the trimester in which the dietary interventions began between the two first order links.

^b^There were important differences in GDM status, ethnicity, and at the trimester in which the dietary interventions began between the two first order links.

^c^Quality of comparisons was assumed as very low because the link order is ≥2.

^d^There were important differences in ethnicity and at the trimester in which the dietary interventions began between the two first order links.

^e^There were important differences at the trimester in which the dietary interventions began between the two first order links.

^f^There were important differences in ethnicity between the two first order links.

^g^There were important differences in pre-pregnancy BMI and at the trimester in which the dietary interventions began between the two first order links.
