# Supplementary material for: The effects of various diets on glycemic outcomes during pregnancy: A systematic review and network meta-analysis
Source: PLoS One. 2017 Aug 3;12(8):e0182095. doi: 10.1371/journal.pone.0182095 (PMC5542432; doi:10.1371/journal.pone.0182095)
Supplement: S5 Table — Abbreviations: CHO, carbohydrate; CIs, confidence intervals; DASH, Dietary Approach to Stop Hypertension; GWG, gestational weight gain; HbA1c, hemoglobin A1c; LGI, low glycemic index; LGL, low glycemic load; MD, mean difference; MUFA, monounsaturated fatty acids; n, sample size. Abbreviations: CHO, carbohydrate; CrI, credible intervals; FG, fasting glucose; GWG, gestational weight gain; LGI, low glycemic index; LGL, low glycemic load; MeD, median difference. (DOCX) [file pone.0182095.s015.docx]

**Table S5.** **Quality of evidence in the mixed dietary comparisons in the fasting glucose analysis.**

|  | **Direct Comparisons** | | **Indirect Comparisons** | | **Overall Network** | |
| --- | --- | --- | --- | --- | --- | --- |
| **Dietary Comparison** | **FG, mmol/L**  **MeD (95% CrIs)** | **Quality of Evidence** | **FG, mmol/L**  **MeD (95% CrIs)** | **Quality of Evidence** | **FG, mmol/L**  **MeD (95% CrIs)** | **Quality of Evidence** |
| **GWG advice provided in both dietary arms** | | | | | | |
| Low-CHO & high-fat diet vs  GWG advice only | -0.60  (-1.00, -0.21) | **⊕⊕⊕⭘**  **MODERATE** | -0.08  (-0.48, 0.32) | **⊕⭘⭘⭘**  **VERY LOW** | -0.35  (-0.63, -0.07) | **⊕⊕⊕⭘**  **MODERATE** |
| LGI/LGL diet vs  GWG advice only | -0.18  (-0.41, 0.04) | **⊕⭘⭘⭘**  **VERY LOW** | -0.71  (-1.20, -0.20) | **⊕⭘⭘⭘**  **VERY LOW** | -0.27 (-0.47, -0.06) | **⊕⭘⭘⭘**  **VERY LOW** |
| LGI/LGL diet vs  Low-CHO & high-fat diet | -0.10  (-0.43, 0.22) | **⊕⭘⭘⭘**  **VERY LOW** | 0.42  (-0.03, 0.86) | **⊕⭘⭘⭘**  **VERY LOW** | 0.08  (-0.18, 0.34) | **⊕⭘⭘⭘**  **VERY LOW** |

**Abbreviations:** CHO, carbohydrate; CrI, credible intervals; FG, fasting glucose; GWG, gestational weight gain; LGI, low glycemic index; LGL, low glycemic load; MeD, median difference.
