# Supplementary material for: The effects of various diets on glycemic outcomes during pregnancy: A systematic review and network meta-analysis
Source: PLoS One. 2017 Aug 3;12(8):e0182095. doi: 10.1371/journal.pone.0182095 (PMC5542432; doi:10.1371/journal.pone.0182095)
Supplement: S6 Table — aInconsistency could not be assessed because only one trial was included. bThere was evidence of high inter-study heterogeneity (I2 = 88%). Further the two included trials showed different effects, one showed protection and the other showed null. cThe included trial(s) failed to achieve its dietary goals and therefore, the contrast of the dietary interventions may be too small to affect HbA1c. dThe effect estimate crosses the minimally important difference (MID) of ±0.3%. eOptimal information size (OIS) was not met. fPublication bias could not be assessed because there were <10 included trials. gNo evidence of inter-study heterogeneity (I2 = 0%). (DOCX) [file pone.0182095.s016.docx]

**Table S6.** **Quality of the evidence in the direct dietary comparisons in the Hb_A1c_ analysis.**

| **Dietary Comparison** | **No of trials  (*n* participants)** | **Hb_A1c_ (%)**  **MD**  **(95% CIs)** | **Risk of Bias** | **Consistency** | **Directness** | **Precision** | **Publication Bias** | **Quality of Evidence** |  |
| --- | --- | --- | --- | --- | --- | --- | --- | --- | --- |
| **GWG advice provided in both dietary arms** | | | | | | | | | |
| High-fibre & low-fat diet vs  Low-CHO & high-fat diet | 1  (20) | -0.80  (-1.98, 0.38) | 0 | 0^a^ | 0 | -2^d,e^ | 0^f^ | **⊕⊕⭘⭘**  **LOW** |  |
| LGI/LGL diet vs  GWG advice only | 2  (103) | 0.01  (-0.05, 0.07) | 0 | -1^b^ | -2^c^ | -1^e^ | 0^f^ | **⊕⭘⭘⭘**  **VERY LOW** |  |
| High unsaturated fat diet vs  GWG advice only | 1  (135) | 0.00  (-0.08, 0.08) | 0 | 0^a^ | -2^c^ | -1^e^ | 0^f^ | **⊕⭘⭘⭘**  **VERY LOW** |  |
| High-MUFA diet vs  GWG advice only | 1  (27) | 0.40  (0.12, 0.68) | 0 | 0^a^ | 0 | -1^d,e^ | 0^f^ | **⊕⊕⊕⭘**  **MODERATE** |  |
| **GWG advice provided in one of the dietary arms** | | | | | | | | | |
| Low-CHO diet & GWG advice vs  High-fibre diet | 1  (28) | -0.50  (-1.87, 0.87) | 0 | 0^a^ | 0 | -2^d,e^ | 0^f^ | **⊕⊕⭘⭘**  **LOW** |  |
| Low-CHO diet & GWG advice vs  Low-CHO diet | 1  (124) | -0.20  (-0.64, 0.24) | 0 | 0^a^ | -2^c^ | -1^d,e^ | 0^f^ | **⊕⭘⭘⭘**  **VERY LOW** |  |
| **GWG advice not provided in any of the dietary arms** | | | | | | | | | |
| DASH-style diet vs  Standard of care | 1  (34) | -0.25  (-2.03, 1.53) | 0 | 0^a^ | 0 | -2^d,e^ | 0^f^ | **⊕⊕⭘⭘**  **LOW** |  |
| LGI diet vs  High-fibre diet | 2  (213) | 0.00  (-0.00, 0.00) | 0 | 0^g^ | -2^c^ | -1^e^ | 0^f^ | **⊕⭘⭘⭘**  **VERY LOW** |  |

**Abbreviations:** CHO, carbohydrate; CIs, confidence intervals; DASH, Dietary Approach to Stop Hypertension; GWG, gestational weight gain; Hb_A1c_, hemoglobin A1c; LGI, low glycemic index; LGL, low glycemic load; MD, mean difference; MUFA, monounsaturated fatty acids; *n*, sample size.

^a^Inconsistency could not be assessed because only one trial was included.

^b^There was evidence of high inter-study heterogeneity (I^2^= 88%). Further the two included trials showed different effects, one showed protection and the other showed null.

^c^The included trial(s) failed to achieve its dietary goals and therefore, the contrast of the dietary interventions may be too small to affect Hb_A1c_.

^d^The effect estimate crosses the minimally important difference (MID) of ±0.3%.

^e^Optimal information size (OIS) was not met.

^f^Publication bias could not be assessed because there were <10 included trials.

^g^No evidence of inter-study heterogeneity (I^2^= 0%).
