# Supplementary material for: The effects of various diets on glycemic outcomes during pregnancy: A systematic review and network meta-analysis
Source: PLoS One. 2017 Aug 3;12(8):e0182095. doi: 10.1371/journal.pone.0182095 (PMC5542432; doi:10.1371/journal.pone.0182095)
Supplement: S7 Table — Abbreviations: CHO, carbohydrate; CIs, confidence intervals; DASH, Dietary Approach to Stop Hypertension; FI, fasting insulin; GWG, gestational weight gain; LGI, low glycemic index; MD, mean difference; MUFA, monounsaturated fatty acids; n, sample size. aDietary comparison was downgraded because the attrition rate of the included trial was considered to have high risk of bias. bInconsistency could not be assessed because only one trial was included. cNo evidence of inter-study heterogeneity (I2 = 0%). dThe included trial(s) failed to achieve its dietary goals and therefore, the contrast of the dietary interventions may be too small to affect FI. eThe effect estimate crosses the minimally important difference (MID) of ±0.5 pmol/L. fOptimal information size (OIS) was not met. gPublication bias could not be assessed because there were <10 included trials. (DOCX) [file pone.0182095.s017.docx]

**Table S7.** **Quality of the evidence in the direct dietary comparisons in the fasting insulin analysis.**

**Abbreviations:** CHO, carbohydrate; CIs, confidence intervals; DASH, Dietary Approach to Stop Hypertension; FI, fasting insulin; GWG, gestational weight gain; LGI, low glycemic index; MD, mean difference; MUFA, monounsaturated fatty acids; *n*, sample size.

| **Dietary Comparison** | **No of trials  (*n* participants)** | **FI, pmol/L**  **MD (95% CIs)** | **Risk of Bias** | **Consistency** | **Directness** | **Precision** | **Publication Bias** | **Quality of Evidence** |  |
| --- | --- | --- | --- | --- | --- | --- | --- | --- | --- |
| **GWG advice provided in both dietary arms** | | | | | | | | | |
| Low-CHO & high-fat diet vs  GWG advice only | 1  (12) | -55.56  (-117.18, 6.06) | 0 | 0^b^ | 0 | -2^e,f^ | 0^g^ | **⊕⊕⭘⭘**  **LOW** |  |
| High-MUFA diet vs  GWG advice only | 1  (27) | 8.96  (-34.62, 52.54) | 0 | 0^b^ | 0 | -2^e,f^ | 0^g^ | **⊕⊕⭘⭘**  **LOW** |  |
| **GWG advice provided in one of the dietary arms** | | | | | | | | | |
| GWG advice only vs  Standard of care | 1  (50) | -25.00  (-46.50, -3.50) | 0^a^ | 0^b^ | 0 | -1^f^ | 0^g^ | **⊕⊕⊕⭘**  **MODERATE** |  |
| **GWG advice not provided in any of the dietary arms** | | | | | | | | | |
| DASH-style diet vs  Standard of care | 2  (65) | -47.60  (-77.34, -17.86) | 0 | 0^c^ | 0 | -1^f^ | 0^g^ | **⊕⊕⊕⭘**  **MODERATE** |  |
| LGI diet vs  High-fibre diet | 1  (92) | 10.80  (-10.66, 32.26) | 0 | 0^b^ | -2^d^ | -2^e,f^ | 0^g^ | **⊕⭘⭘⭘**  **VERY LOW** |  |

^a^Dietary comparison was downgraded because the attrition rate of the included trial was considered to have high risk of bias.

^b^Inconsistency could not be assessed because only one trial was included.

^c^No evidence of inter-study heterogeneity (I^2^= 0%).

^d^The included trial(s) failed to achieve its dietary goals and therefore, the contrast of the dietary interventions may be too small to affect FI.

^e^The effect estimate crosses the minimally important difference (MID) of ±0.5 pmol/L.

^f^Optimal information size (OIS) was not met.

^g^Publication bias could not be assessed because there were <10 included trials.
