# Supplementary material for: The effects of various diets on glycemic outcomes during pregnancy: A systematic review and network meta-analysis
Source: PLoS One. 2017 Aug 3;12(8):e0182095. doi: 10.1371/journal.pone.0182095 (PMC5542432; doi:10.1371/journal.pone.0182095)
Supplement: S8 Table — Abbreviations: CHO, carbohydrate; CIs, confidence intervals; DASH, Dietary Approach to Stop Hypertension; GWG, gestational weight gain; HOMA-IR, homeostatic model assessment for insulin resistance; LGI, low glycemic index; MD, mean difference; n, sample size. aInconsistency could not be assessed because only one trial was included. bThe effect estimate crosses the minimally important difference (MID) of ±1 unit. cOptimal information size (OIS) was not met. dPublication bias could not be assessed because there were <10 included trials. eNo evidence of inter-study heterogeneity (I2 = 0%). fThe included trial failed to achieve its dietary goals and therefore, the contrast of the dietary interventions may be too small to affect HOMA-IR. (DOCX) [file pone.0182095.s018.docx]

**Table S8.** **Quality of the evidence in the direct dietary comparisons in the HOMA-IR analysis.**

| **Dietary Comparison** | **No of trials  (*n* participants)** | **HOMA-IR**  **MD**  **(95% CIs)** | **Risk of Bias** | **Consistency** | **Directness** | **Precision** | **Publication Bias** | **Quality of Evidence** |
| --- | --- | --- | --- | --- | --- | --- | --- | --- |
| **GWG advice provided in both dietary arms** | | | | | | | | |
| Low-CHO & high-fat diet vs  GWG advice only | 1  (12) | -2.10  (-5.20, 1.00) | 0 | 0^a^ | 0 | -2^b,c^ | 0^d^ | **⊕⊕⭘⭘**  **LOW** |
| **GWG advice not provided in any of the dietary arms** | | | | | | | | |
| DASH-style diet vs  Standard of care | 2  (65) | -1.90  (-3.08, -0.72) | 0 | 0^e^ | 0 | -1^b,c^ | 0^d^ | **⊕⊕⊕⭘**  **MODERATE** |
| LGI diet vs  High-fibre diet | 1  (92) | -0.10  (-0.34, 0.14) | 0 | 0^a^ | -2^f^ | -1^c^ | 0^d^ | **⊕⭘⭘⭘**  **VERY LOW** |

**Abbreviations:** CHO, carbohydrate; CIs, confidence intervals; DASH, Dietary Approach to Stop Hypertension; GWG, gestational weight gain; HOMA-IR, homeostatic model assessment for insulin resistance; LGI, low glycemic index; MD, mean difference; *n*, sample size.

^a^Inconsistency could not be assessed because only one trial was included.

^b^The effect estimate crosses the minimally important difference (MID) of ±1 unit.

^c^Optimal information size (OIS) was not met.

^d^Publication bias could not be assessed because there were <10 included trials.

^e^No evidence of inter-study heterogeneity (I^2^= 0%).

^f^The included trial failed to achieve its dietary goals and therefore, the contrast of the dietary interventions may be too small to affect HOMA-IR.
