# Supplementary material for: The effects of various diets on glycemic outcomes during pregnancy: A systematic review and network meta-analysis
Source: PLoS One. 2017 Aug 3;12(8):e0182095. doi: 10.1371/journal.pone.0182095 (PMC5542432; doi:10.1371/journal.pone.0182095)
Supplement: S2 File — (PDF) [file pone.0182095.s020.pdf]

PROSPERO International prospective register of systematic reviews

Review title and timescale

1 Review title
Give the working title of the review. This must be in English. Ideally it should state succinctly the interventions or exposures being reviewed and the associated health or social problem being addressed in the review.
The Effects of Dietary Patterns on Glycaemic Management in Pregnant Women: A Systematic Review and Network Meta-Analysis of Randomized Controlled Trials.

2 Original language title
For reviews in languages other than English, this field should be used to enter the title in the language of the review. This will be displayed together with the English language title.

3 Anticipated or actual start date
Give the date when the systematic review commenced, or is expected to commence.
01/09/2014

4 Anticipated completion date
Give the date by which the review is expected to be completed.
31/12/2015

5 Stage of review at time of this submission
Indicate the stage of progress of the review by ticking the relevant boxes. Reviews that have progressed beyond the point of completing data extraction at the time of initial registration are not eligible for inclusion in PROSPERO. This field should be updated when any amendments are made to a published record.

The review has not yet started ×

Review stage	Started	Completed
Preliminary searches	Yes	No
Piloting of the study selection process	Yes	No
Formal screening of search results against eligibility criteria	Yes	No
Data extraction	Yes	No
Risk of bias (quality) assessment	No	No
Data analysis	No	No

Provide any other relevant information about the stage of the review here.

Review team details

6 Named contact
The named contact acts as the guarantor for the accuracy of the information presented in the register record.
Russell de Souza

7 Named contact email
Enter the electronic mail address of the named contact.
rdesouz@mcmaster.ca

8 Named contact address
Enter the full postal address for the named contact.
#3210- 1280 Main Street West, Hamilton, Ontario, CANADA L8S 4L8,

9 Named contact phone number
Enter the telephone number for the named contact, including international dialing code.
1 905 525 9140 ext.22109

10 Organisational affiliation of the review
Full title of the organisational affiliations for this review, and website address if available. This field may be completed as 'None' if the review is not affiliated to any organisation.

Chanchlani Research Centre, McMaster University

Website address:

11 Review team members and their organisational affiliations

Give the title, first name and last name of all members of the team working directly on the review. Give the organisational affiliations of each member of the review team.

Title	First name	Last name	Affiliation
Ms	Vanessa	Ha	McMaster University
Mr	Ashley	Bonner	McMaster University
Mr	Jaynendr	Jadoo	None
Dr	Joseph	Beyene	McMaster University
Dr	Sonia	Anand	McMaster University
Dr	Russell	de Souza	McMaster University

12 Funding sources/sponsors

Give details of the individuals, organizations, groups or other legal entities who take responsibility for initiating, managing, sponsoring and/or financing the review. Any unique identification numbers assigned to the review by the individuals or bodies listed should be included.

Canadian Institutes of Health Research (CIHR)

13 Conflicts of interest

List any conditions that could lead to actual or perceived undue influence on judgements concerning the main topic investigated in the review.

Are there any actual or potential conflicts of interest?

None known

14 Collaborators

Give the name, affiliation and role of any individuals or organisations who are working on the review but who are not listed as review team members.

Title	First name	Last name	Organisation details

Review methods

15 Review question(s)

State the question(s) to be addressed / review objectives. Please complete a separate box for each question.

Primary Question: What is the comparative effectiveness of various dietary patterns on glycaemic management in pregnant women?

Secondary Question: Which of the reviewed diets is likely to be the best for glycaemic control in pregnant women?

16 Searches

Give details of the sources to be searched, and any restrictions (e.g. language or publication period). The full search strategy is not required, but may be supplied as a link or attachment.

MEDLINE, EMBASE, Cochrane Central Register of Controlled Trials, and manual search of published studies will be searched to identify eligible studies. No restriction on language and year of publication will be placed.

17 URL to search strategy

If you have one, give the link to your search strategy here. Alternatively you can e-mail this to PROSPERO and we will store and link to it.

I give permission for this file to be made publicly available

Yes

18 Condition or domain being studied

Give a short description of the disease, condition or healthcare domain being studied. This could include health and wellbeing outcomes.

We will review glycaemic outcomes including fasting blood glucose (FBG), fasting blood insulin (FBI), Hemoglobin A1c (HbA1c), and Homeostatic Model Assessment- Insulin Resistance (HOMA-IR).

19 Participants/population

Give summary criteria for the participants or populations being studied by the review. The preferred format includes details of both inclusion and exclusion criteria.

Pregnant women regardless of their diabetes status will be included in our review. Dietary studies with non-pregnant women will be excluded.

20 Intervention(s), exposure(s)

Give full and clear descriptions of the nature of the interventions or the exposures to be reviewed

To be included in our review, studies must have studied dietary patterns on fasting blood glucose (FBG), fasting blood insulin (FBI), Hemoglobin A1c (HbA1c), or Homeostatic Model Assessment- Insulin Resistance (HOMA-IR). Studies that studied single nutrients or foods and/or on other outcomes other than glycaemic outcomes will be excluded.

21 Comparator(s)/control

Where relevant, give details of the alternatives against which the main subject/topic of the review will be compared (e.g. another intervention or a non-exposed control group).

The comparator must be another dietary pattern or standard of care (eg. generic dietary advice or no formal dietary advice given). Studies will be excluded if any other comparator was used.

22 Types of study to be included initially

Give details of the study designs to be included in the review. If there are no restrictions on the types of study design eligible for inclusion, this should be stated.

Only published randomized controlled trials reporting an explicit comparison between 2 or more dietary patterns will be included in our review. Co-intervention studies (eg. dietary pattern + medication or dietary pattern + exercise) are allowed as along as the effect of the dietary patterns can be isolated. No restriction on language and year of publication will be placed.

23 Context

Give summary details of the setting and other relevant characteristics which help define the inclusion or exclusion criteria.

Studies must have a follow-up duration of at least 4-weeks.

24 Primary outcome(s)

Give the most important outcomes.

Fasting Blood Glucose (FBG)

Give information on timing and effect measures, as appropriate.

25 Secondary outcomes

List any additional outcomes that will be addressed. If there are no secondary outcomes enter None.

Fasting blood insulin (FBI), Hemoglobin A1c (HbA1c), and Homeostatic Model Assessment- Insulin Resistance (HOMA-IR).

Give information on timing and effect measures, as appropriate.

26 Data extraction, (selection and coding)

Give the procedure for selecting studies for the review and extracting data, including the number of researchers involved and how discrepancies will be resolved. List the data to be extracted.

The title and abstract of studies identified from our electronic and manual search will be assessed for eligibility. This will be followed by a full text assessment if the study had passed the title/abstract screening. Two independent reviewers will extract study characteristics and data onto a standardized proforma. Discrepancies will be identified and resolved through discussion and if necessary with Dr. Russell de Souza. Missing data will be requested from study authors and/or imputed using standard formulae.

27 Risk of bias (quality) assessment

State whether and how risk of bias will be assessed, how the quality of individual studies will be assessed, and whether and how this will influence the planned synthesis.

Two independent reviewers will assess the risk of bias (ROB) in each of the included studies. The assesment from ROB will be used to help inform our assessment for the quality of the evidence for each outcome, which we will assess using Grading of Recommendations Assessment, Development and Evaluation (GRADE). Disagreements between will be resolved by discussion and if necessary with Dr. Russell de Souza.

28 Strategy for data synthesis

Give the planned general approach to be used, for example whether the data to be used will be aggregate or at the level of individual participants, and whether a quantitative or narrative (descriptive) synthesis is planned. Where appropriate a brief outline of analytic approach should be given.

The network geometry for each outcome will be assessed for feasibility of conducting a network meta-analysis. Factors that will be examined include diversity, co-occurrence, transitivity and consistency. A Bayesian framework will be used to perform a random-effects network meta-analysis, where the pooled effect estimate will be expressed as mean differences with 95% credible intervals (CrI).The prior distribution for treatment effects will be assumed to be minimally informative and analyses will be performed using Markov-Chain Monte-Carlo Methods where convergence will be evaluated using Gelman-Rubin statistics. Ranking of the effectiveness of dietary patterns will be evaluated using surface under the cumulative ranking (SUCRA).Heterogeneity will be assessed by the Cohran Q-statistics and quantified by the I2.

29 Analysis of subgroups or subsets

Give any planned exploration of subgroups or subsets within the review. 'None planned' is a valid response if no subgroup analyses are planned.

To explore potential sourcesof heterogeneity, we will conduct subgroup analyses on diabetes status (gestational diabetes, pre-pregnancy type 2 diabets, no diabetes), pre-pregnancy body weight, gestation weight gain, and ethniciy, where there are >10 trials for a particular outcome.

Review general information

30 Type of review

Select the type of review from the drop down list.

Intervention

31 Language

Select the language(s) in which the review is being written and will be made available, from the drop down list. Use the control key to select more than one language.

English

Will a summary/abstract be made available in English?

Yes

32 Country

Select the country in which the review is being carried out from the drop down list. For multi-national collaborations select all the countries involved. Use the control key to select more than one country.

Canada

33 Other registration details

Give the name of any organisation where the systematic review title or protocol is registered together with any unique identification number assigned. If extracted data will be stored and made available through a repository such as the Systematic Review Data Repository (SRDR), details and a link should be included here.

34 Reference and/or URL for published protocol

Give the citation for the published protocol, if there is one.

Give the link to the published protocol, if there is one. This may be to an external site or to a protocol deposited with CRD in pdf format.

I give permission for this file to be made publicly available

Yes

35 Dissemination plans

Give brief details of plans for communicating essential messages from the review to the appropriate audiences.

We will publish a manuscript of the review in a peer-reviewed journal and dessiminate the results in national and international conferences.

Do you intend to publish the review on completion?

36 Keywords
Give words or phrases that best describe the review. (One word per box, create a new box for each term)
Gestational Diabetes

Glycaemic Management

Dietary Patterns

Systematic Review

Network Meta-Analysis

Pregnant Women

Glycaemic Outcome

Maternal Health

37 Details of any existing review of the same topic by the same authors
Give details of earlier versions of the systematic review if an update of an existing review is being registered, including full bibliographic reference if possible.

38 Current review status
Review status should be updated when the review is completed and when it is published.
Ongoing

39 Any additional information
Provide any further information the review team consider relevant to the registration of the review.

40 Details of final report/publication(s)
This field should be left empty until details of the completed review are available.
Give the full citation for the final report or publication of the systematic review.
Give the URL where available.
